# Improving Exploitation of Whole Genome Sequencing Data for Public Health, Forensic Microbiology and Biosafety

**DOI:** 10.1016/j.ebiom.2015.11.011

**Published:** 2015-11-10

**Authors:** Paola Pilo

**Affiliations:** Institute of Veterinary Bacteriology, Vetsuisse Faculty, University of Bern, Laenggassstrasse 122, 3012, Bern, Switzerland

*Bacillus anthracis*, a Gram-positive spore forming rod-shaped bacterium, is the causative agent of anthrax. This zoonotic disease principally affects herbivores but *B. anthracis* can also infect other livestock and wildlife species (WHO, 1998). Human cases mainly occur sporadically and infections mostly follow direct or indirect contact with contaminated animal products. Three major forms of human anthrax are described in the literature: cutaneous, gastrointestinal and pulmonary. All these forms can successively lead to septicemia and meningitis. The development of a specific clinical manifestation is dependent upon the route of infection (WHO, 1998). Recently, growing concern was raised by reports describing a new form of anthrax found in heroin users resulting from the injection of the drug contaminated with spores of *B. anthracis* (Hanczaruk et al., 2014). Symptoms of injectional anthrax (IA) are more severe than those observed in classical cutaneous anthrax and clinical outcome is poor (Berger et al., 2014). Two outbreaks of IA occurred in 2009–2010 (UK, Germany) and in 2012–2013 (Denmark, France, Germany, UK), respectively (Berger et al., 2014). The subsequent investigations performed to determine the source of infection involved multi-disciplinary teams and turned out to be a complicated task as a consequence of the illegal aspects related to heroin production, distribution and use (Team NAOC, 2011). Among the large amount of data collected during these investigations, genotyping of strains of *B. anthracis* was fundamental (Price et al., 2012).

The recent improvements in the field of whole genome sequencing (WGS) opened up a large panel of applications for bacterial research and diagnostics. However, WGS generates an enormous quantity of data and it is essential to maximize exploitation of the produced information for epidemiological and evolutionary studies. Public health authorities need typing of pathogenic bacterial isolates to plan and implement disease surveillance and rapid outbreak response (Sabat et al., 2013). This facilitates a better understanding of the dynamics of epidemic events and improves traceability of sources of infection. From this point of view, WGS allows a higher resolution for typing bacteria than the previously available methods based on molecular biology techniques (Bertelli and Greub, 2013). Moreover, the comparison of genomes and consequent assessment of variation among related and unrelated isolates enables the selection of adequate genetic markers for rapid screening of isolates.

In this issue of EBioMedicine, Keim and colleagues (Keim et al., 2015) describe an efficient and well-designed study demonstrating how to take full advantage of genomic data focusing principally on single nucleotide polymorphisms. They analyzed the genomes of 60 isolates collected in Europe from IA cases between 2000 and 2013 using a standardized computational pipeline. Additionally, they screened two large strain collections consisting of 1293 isolates. These investigations led to the identification of two major related clusters of *B. anthracis* isolates associated with IA cases: G-I and G-II. All isolates from the 2009–2010 outbreak (Germany, UK) clustered within the G-I group, while isolates from 2012 to 2013 (Denmark, Germany, UK) and the isolate from 2000 (Norway) clustered in the G-II group (Keim et al., 2015). Consequently, this study suggests the occurrence of at least two contaminations events from close geographical areas. Previous results indicated that contamination of heroin likely arose during drug transport/distribution because of the close genetic relationships of IA isolates to 2 strains isolated in Turkey, which is presumably a transit country for heroin distribution in Western Europe (Price et al., 2012). The screening of the *B. anthracis* strain collections identified five additional strains related to the IA cases isolated in the US (Keim et al., 2015), probably originating from imported goods but with unknown origin stressing the necessity of extensive sampling and detailed documentation of strains. This is extremely relevant when comparing strains in a global and historical context. A supplementary finding of interest in this study is the observation of genetic variability among isolates within single patients. The phylogenetic analyses performed by Keim and colleagues indicate that the probable cause of this diversity is the infecting spores (Keim et al., 2015). Co-infection with more than one spore genotype was also suggested by Beyer and Turnbull in animals (Beyer and Turnbull, 2013). However, further in vitro and in vivo experiments in animal models would warrant particular attention to better understand population dynamics of *B. anthracis* within hosts and to confirm or refute hypotheses based on observations made in the case of natural infections.

Beyond the importance of these results for the *B. anthracis* field, this study highlights four indispensable points to increase efficiency and general knowledge resulting from the WGS: 1) standardization of computational pipelines, 2) accessibility to large strain collections, 3) availability of complete information about isolates and 4) coupling data obtained from natural cases to experimental assays. Furthermore, the massive work performed in *B. anthracis* genomics made this bacterium an excellent model organism and this approach should be further applied to other pathogenic bacteria to efficiently exploit genomic data for evolutionary studies, public health, forensic microbiology and biosafety issues.

## Conflict of Interest

The author declares no conflict of interest.
